# Donor-Cell Origin High-Risk Myelodysplastic Syndrome Synchronous with an Intracranial Meningioma-Like Tumor, 8 Years after Allogeneic Hematopoietic Stem Cell Transplantation for Chronic Lymphocytic Leukemia

**DOI:** 10.1155/2017/9674385

**Published:** 2017-08-03

**Authors:** G. Brás, C. Pinho-Vaz, A. Campos

**Affiliations:** Serviço de Transplante de Medula Óssea, Instituto Português de Oncologia do Porto, Porto, Portugal

## Abstract

Secondary neoplasias are well known consequences of radiotherapy or chemotherapy for a primary cancer. In this report, we describe two rare secondary neoplasias occurring in the same patient: a meningioma-like intracranial tumor and high-risk myelodysplastic syndrome (MDS) of donor-cells origin, both diagnosed simultaneously, 8 years after an allogeneic hematopoietic stem cell transplantation (allo-HSCT) for chronic lymphocytic leukemia (CLL). Due to an engraftment failure during the first allo-HSCT of a matched related donor for CLL treatment, the salvage treatment was a second allo-HSCT. At the moment of meningioma-like tumor diagnosis, the patient was pancytopenic due to high-risk MDS, so it was decided to postpone a surgical intervention until hematological improvement. For the high-risk MDS of donor-cells origin the chosen treatment was induction with intensive chemotherapy. Due to refractory disease, the patient was treated with 5-azacitidine and donor-lymphocytes infusion with no response and, finally, a third allo-HSCT of a matched unrelated donor was performed. The patient died 6 months after the third allo-HSCT, in cytogenetic remission but without hematological recovery, due to an intracranial hemorrhage with origin in the meningioma-like tumor.

## 1. Introduction

Allogeneic Hematopoietic Stem Cell Transplantation (Allo-HSCT) is the only curative approach for the majority of hematooncological diseases. The major limitations of the Allo-HSCT are the short and long-term toxicities related to the procedure. The recent advances in transplantation techniques and supportive care were responsible for a decrease in therapy-related mortality and improvement of overall survival (OS). Increasing the OS leads us to the problematic secondary neoplasias. During the first year after HSCT, apart from relapse, posttransplant lymphoproliferative disorder is the most frequent secondary malignancy with a cumulative incidence between 0,6 and 1,4% in allo-HSCT and extremely rare in autologous HSCT (auto-HSCT). In terms of secondary solid cancers, the cumulative incidence increases over time: 1-2% at 5 years; 2–6% at 10 years, and 3–15% at 15 years. Myelodysplastic syndrome (MDS) and acute myeloid leukemia (AML) have an incidence of 5–15% at 5 years after auto-HSCT, but extremely rare after allo-HSCT [1]. The main risk factors for MDS/AML in patients previously treated for hematological diseases are older age prior to HSCT, the type and intensity of pre-HSCT chemotherapy, and total-body irradiation (TBI) in allo-HSCT conditioning [2]. This report describes two rare events in the same patient: donor-cell origin MDS/leukemia and a meningioma-like intracranial tumor, both diagnosed simultaneously, 8 years after allo-HSCT. In a large retrospective survey of the European Group for Blood and Marrow Transplantation (EBMT), the estimated incidence of donor-cell origin MDS/leukemia after allo-HSCT was less than 1% [3]. The true incidence of meningioma after allo-HSCT is unknown. It has been more frequently described in children cancer survivors exposed to cranial radiotherapy [4]. In a retrospective cohort of acute lymphoblastic leukemia (ALL) childhood survivors, brain tumors were the most prevalent secondary solid cancer and 89% of those patients were exposed to cranial irradiation. In this cohort, the prevalence of meningiomas was 3,4% with a median time for presentation, since ALL diagnosis, of 16 (12–18) years [5]. Intracranial granulocytic sarcomas are an important differential diagnosis with meningiomas, in patients with concurrent acute myeloid leukemia. In a retrospective study of 21 reported cases of intracranial myeloid sarcoma, 11 (52,4%) presented with meningioma-like lesions [6].

## 2. Case Report

A 57-year-old Caucasian male was diagnosed with chronic lymphocytic leukemia (CLL), stage II-B in Rai-Binet System, and unknown cytogenetic risk, in 1998. The CLL was refractory to fludarabine and cyclophosphamide (FC). In September of 2001, an allo-HSCT of a matched related donor (brother) was performed with reduced-intensity conditioning (RIC) of fludarabine and busulfan (FluBu). The acute graft-versus-host disease (aGVHD) prophylaxis was cyclosporine (Csp) and mycophenolate mofetil (MMF). Engraftment failure occurred and a second allo-HSCT of the same donor after RIC with fludarabine and cyclophosphamide plus in vivo lymphodepletion with alemtuzumab was performed. The aGVHD prophylaxis was Csp and MMF again. After successful engraftment and hematological recovery, bone marrow evaluation confirmed complete remission (CR). During the posttransplant follow-up period, neither aGVHD nor chronic GHVD (cGVHD) was observed.

**Figure 1:**
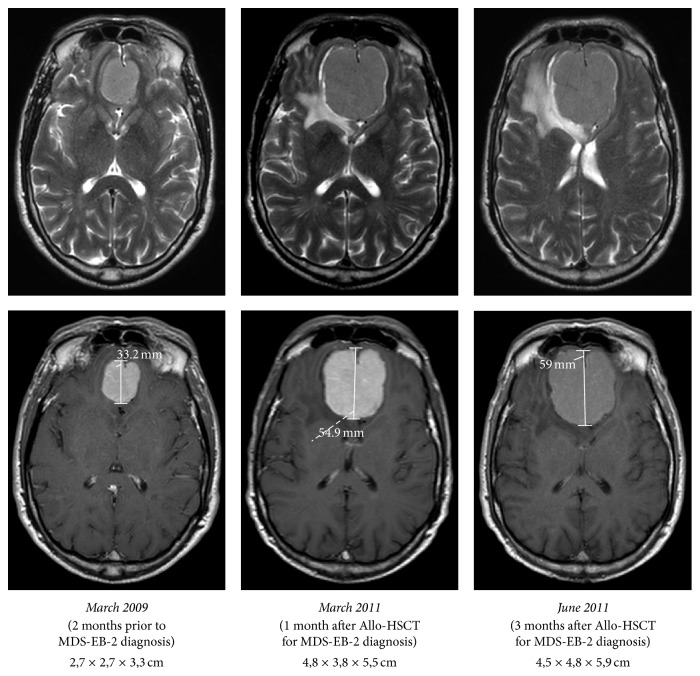
Meningioma-like tumor evolution since diagnosis until patient death.

In March of 2009, due to headache and behavioural alterations, a cerebral magnetic resonance imaging (MRI) was performed and showed an intracranial extra-axial expansive lesion in the anterior cranial fossa measuring 2,7 × 2,7 × 3,3 cm of transversal, cranial-caudal, and anterior-posterior diameters, respectively, suggestive of olfactory groove meningioma (Figure 1). No cerebrospinal fluid evaluation (cytological or immunophenotypical) was made.

During evaluation for neurosurgery, in May of 2009, after almost 8 years in CR for CLL, the patient presented with pancytopenia. A diagnosis of myelodysplastic syndrome with excess blasts-2 (MDS-EB-2) was made based on a bone marrow smear with dysplastic features, blast count of 11%, and karyotype with monosomy 7 in 14 of 20 metaphases. Chimerism analysis by polymerase chain reaction of short tandem repeats (STR-PCR) showed full-donor chimerism in all lineages, which confirmed the donor-cell origin for the MDS. To investigate occult MDS, the donor bone marrow was evaluated and showed no dysplastic features or cytogenetic abnormalities. The donor is currently free of any hematological disease.

Neurosurgical intervention was postponed until resolution of the hematological disease. The MDS-EB-2 was resistant to intensive chemotherapy (cytarabine, daunorubicin, and Csp). Between October of 2009 and February of 2010, the patient went on second-line treatment with 5-azacitidine, abandoned at the end of 4 cycles due to absence of hematological and cytogenetical response. Between May and August of 2010, donor lymphocyte infusions (DLI) were performed twice, with CD3 cells doses of 1 × 10^7^/kg and 1 × 10^8^/kg, respectively, with no response. In January of 2011, a matched unrelated donor (MUD) was identified, which allowed a third allo-HSCT in February of 2011. Prior to the HSCT, the patient was pancytopenic with a bone marrow blast count of 5,5% and a karyotype with monosomy 7. The chosen conditioning regimen was fludarabine and melphalan plus in vivo lymphodepletion with alemtuzumab. The aGVHD prophylaxis was tacrolimus and MMF. In March of 2011, within 1 month of allo-HSCT, the meningioma-like lesion was revaluated by MRI and there was a significant increase in its dimensions (5,5 cm × 3,8 cm cranial-caudal and transversal diameters), associated with oedema and mass effect (Figure 1). In May of 2011, 3 months after allo-HSCT, the patient was in cytogenetic remission, with full-donor chimerism of all lineages in peripheral blood and bone marrow, but with no hematological recovery (hemoglobin 86 g/L, leucocytes 2,7 × 10^9^/L, neutrophils 2,2 × 10^9^/L, and platelets 22 × 10^9^/L). Cytomegalovirus (CMV) reactivation was also diagnosed and daily ganciclovir was started.

In June of 2011, the patient complained of headaches again. The meningioma-like lesion was reassessed by MRI, which showed an increase in size (4,5 × 4,8 × 5,9 cm of major diameters), in relation to intratumor hemorrhage, worsening of oedema, and mass effect (Figure 1). The neurosurgery group decision was conservative management until hematological recovery, given the risk of carrying out surgery with low platelets number. In July of 2011, the patient was hospitalized due to altered mental status and died of intracranial hemorrhage in the meningioma-like tumor.

## 3. Discussion

In terms of etiology for donor-cell MDS/leukemia, the possible explanations are divided into two groups: donor-cell factors and host factors [7]. For donor-cell factors, the high proliferative demands after HSCT might increase the likelihood of replication errors, mutations, and telomeres shortening, ensuing clonal evolution of engrafted donor cells [7, 8]. The escape to immunosurveillance in posttransplant period, due to immunosupression, might be an explanation for the advantage and persistence of the abnormal clone. The exposure to chemotherapy prior to HSCT, namely, in conditioning, has been considered a possible risk factor to induce clonal changes in donor cells [8]. Another hypothesis is the transfer of occult leukemic cells or cells with mutations (clonal hematopoiesis) from the donor, with potential to evolve to MDS/leukemia [9, 10]. In terms of host factors, the bone marrow microenvironment suffers important changes after conditioning, favouring growth, and survival of abnormal clones: (a) chromosomal abnormalities were described in nonhematopoietic cells from the bone marrow of MDS and AML patients; (b) stromal cells of patients with MDS and AML can be permanently activated, with a pattern of cytokine and growth-factors expression that promotes proliferative advantage of abnormal clones [9]. The majority of donor-cell origin leukemias are acute myeloid leukemia, even when the previous disease is of lymphoid origin and this fact raises the hypothesis of an imbalance favouring the myeloid abnormal clonal differentiation, after HSCT [8]. The donor was evaluated to exclude occult hematological disease. He was evaluated by morphology and karyotype. Mutational studies were not performed, so we are unable to exclude a case of occult donor clonal hematopoiesis.

The first in vivo reports of donor-cell MDS/leukemia were made by cytogenetic evaluation of sex-mismatched allo-HSCT, showing abnormalities in a karyotype of different sex [11]. A few years later, the development of restriction fragment length polymorphisms (RFLP) techniques was the major contributor to diagnose donor-cell MDS/leukemia of donor/recipient sex matched allo-HSCT [12]. Nowadays, the STR genotyping has been the most widely used technique to determine the postengraftment chimerism status and the origin of relapsing MDS/leukemia in this type of allo-HSCT [3, 13]. In allo-HSCT with donor/recipient sex match, as this particular report, our centre uses STR-PCR because it is highly sensitive and fast and detects polymorphic regions distinctive of each individual, independent of sex-mismatching.

The treatment of donor-cell MDS/leukemia is also subject of debate. In a multicentre retrospective study, from 14 patients with donor-cell leukemia, 9 were treated with intensive chemotherapy, and CR was achieved in 6 (66,7%) patients [3]. Only one patient had monosomy 7 at the moment of donor-cell leukemia diagnosis and was in progression after rescue intensive chemotherapy, like our patient. Hypomethylating agents (HMA) have been used in MDS/AML relapsing after allo-HSCT. Two possible approaches have been proposed: HMA alone or followed by DLI [14, 15]. In our report, we decided to use 5-azacitidine alone. No response was achieved after 4 cycles. DLI was performed, not in a sequential strategy after HMA, but in context of refractory disease after HMA. There are no prospective and randomized trials comparing intensive chemotherapy versus HMA in relapse after allo-HSCT. In a retrospective cohort, the use of conventional salvage intensive chemotherapy, preferentially followed by DLI, was associated with better outcomes in terms of response and survival than HMA [16]. There is scarce data about the efficacy of DLI in donor-cell MDS/leukemia. In a single report, DLI was successfully used in donor-cell MDS and the rationale was the graft-versus-leukemia (GVL) effect of DLI, even against cells of the same donor [17]. The malignant cells probably acquire neoantigens during the transformation process that enables an immunological response. Additionally, lymphocytes produce IL-6, a cytokine that plays an important role in hematopoiesis stimulation to promote hematological recovery [17]. This report differs from ours in terms of disease characteristics: the blast count was 1% and the karyotype was normal. The only approach that led to a response, in our report, was a new allo-HSCT from a different donor. The rationale to choose a different donor was to potentiate the GVL effect against an aggressive disease that was resistant to conventional chemotherapy, HMA, and DLI. Although the patient did not achieve hematological recovery he was in complete cytogenetic remission with full-donor chimerism of all lineages in bone marrow and peripheral blood. The lack of hematological recovery might be explained by CMV reactivation or by an adverse microenvironment for a full engraftment, due to previous chemotherapy exposure or MDS itself.

Secondary brain tumors are rare events after bone marrow transplantation [2]. Meningiomas are tumors of the meninges that occur sporadically with not known aetiology. The radiation exposure during cancer treatment (cranial radiotherapy or TBI in conditioning for allo-HSTC) has been recognized as the main risk factor for meningioma in cancer survivors [4]. Intrathecal methotrexate has also been implicated as risk factor [18]. In our report, there is no exposure to high doses of radiation or intrathecal methotrexate. There is no published data showing a relation between systemic chemotherapy and meningioma risk. Another hypothesis to be considered is the possibility that the meningioma-like lesion was actually a granulocytic sarcoma, due to a synchronous diagnosis with the MDS-EB-2. In a retrospective study, of 21 reported cases of intracranial myeloid sarcoma, 11 (52,4%) presented with meningioma-like lesions, and from those, 7 presented in association with vasogenic oedema [6]. There is a report of a granulocytic sarcoma mimicking a falx meningioma [19]. In that particular case, the diagnosis was confirmed after surgery and the cerebrospinal liquid analysis was negative for leukemic cells. In our report we were unable to perform histological diagnosis of the meningioma-like lesion. The lesion, apparently, remained stable between 2009 and 2011, period of the rescue intensive chemotherapy, 5-azacitidine, and DLI. The increase in tumor mass one month after allo-HSCT was due to intratumor hemorrhage, probably in relation to thrombocytopenia. Considering that central nervous system myeloid sarcomas may mimic meningioma lesions, efforts should be made to biopsy meningioma-like lesions in patients with myeloid malignancies. In a prospective study of postmortem evaluation of neuropathological complications after bone marrow transplantation, of 371 deaths in a cohort of 845 transplants, 180 autopsies with brain evaluation were performed. Central nervous system abnormalities were found in about one of every 2.3 deaths but brain tumors were not found, even in patients exposed to TBI [20]. In a retrospective cohort of 111 patients who underwent autopsy after HSCT, there were no cases of brain tumor reported. The premortem and postmortem diagnosis differed in 26% of cases, and the autopsy provided important clues to change the therapeutic approach in 4% [21]. Considering these data, the role of autopsy after HSCT might be controversial in cases of clinical obvious cause of death; nevertheless, postmortem diagnosis can be important in cases of unknown cause of death and cases with atypical and rare clinical presentation, like this report. The correlation between image and histology, even in postmortem evaluation, might be important to improve our knowledge about the radiologic patterns that different types of secondary brain tumors might present.
